# Health and Human Rights in Karen State, Eastern Myanmar

**DOI:** 10.1371/journal.pone.0133822

**Published:** 2015-08-26

**Authors:** William W. Davis, Luke C. Mullany, Eh Kalu Shwe Oo, Adam K. Richards, Vincent Iacopino, Chris Beyrer

**Affiliations:** 1 Center for Public Health and Human Rights, Bloomberg School of Public Health, Baltimore, Maryland, United States of America; 2 Karen Department of Health and Welfare, Mae Sot, Thailand; 3 Division of General Internal Medicine and Health Services Research, University of California Los Angeles, Los Angeles, California, United States of America; 4 Community Partners International, 2550 Ninth St. Suite 111, Berkeley, California, 94710, United States of America; 5 Physicians for Human Rights, New York City, New York, United States of America; 6 University of Minnesota Medical School, Minneapolis, Minnesota, United States of America; 7 Human Rights Center, University of California, Berkeley, California, United States of America; Queensland University of Technology, AUSTRALIA

## Abstract

**Background:**

Decades of conflict in eastern Myanmar have resulted in high prevalence of human rights violations and poor health outcomes. While recent ceasefire agreements have reduced conflict in this area, it is unknown whether this has resulted in concomitant reductions in human rights violations.

**Methods and Findings:**

We conducted a two-stage cluster survey of 686 households in eastern Myanmar to assess health status, access to healthcare, food security, exposure to human rights violations and identification of alleged perpetrators over the 12 months prior to January 2012, a period of near-absence of conflict in this region. Household hunger (FANTA-2 scale) was moderate/high in 91 (13.2%) households, while the proportion of households reporting food shortages in each month of 2011 ranged from 19.9% in December to 47.0% in September, with food insecurity peaking just prior to the harvest. Diarrhea prevalence in children was 14.2% and in everyone it was 5.8%. Forced labor was the most common human rights violation (185 households, 24.9%), and 210 households (30.6%) reported experiencing one or more human rights violations in 2011. Multiple logistic regression analysis identified associations between human rights violations and poor health outcomes.

**Conclusion:**

Human rights violations and their health consequences persist despite reduced intensity of conflict in eastern Myanmar. Ceasefire agreements should include language that protects human rights, and reconciliation efforts should address the health consequences of decades of human rights violations.

## Background

Karen state, in eastern Myanmar, has experienced six decades of low-intensity conflict that has had severe impacts on the civilian population. Direct effects on the population including forced displacement, pillaged food stores, injury from violence and forced labor, [1–7] while indirect effects of the war include poor transportation infrastructure, poor supply chains for clinics, and increased risk for healthcare providers. [2–11]

Numerous prior studies have demonstrated strong links between human rights violations in this region and population-based health indicators. They found that forced displacement was associated with child malnutrition and child mortality, that theft and destruction of food supply was associated with malaria parasitemia and child malnutrition, and that those exposed to human rights violations had severely curtailed access to essential maternal health interventions. [12,13]

Although these studies were done in conflict areas, reports from Myanmar suggest that similar human rights violations can and do occur in areas of low or no conflict. A 2011 survey from the predominantly non-conflict Chin State in western Myanmar found a 91% prevalence of forced labor, with the Myanmar army responsible for a majority of the violations. [14] Qualitative reports from community-based human rights groups suggest that militarization, or the presence of armed groups, regardless of combat status, results in human rights violations in Karen state. [15–17]

Official peace negotiations began in Karen state 2011, and a preliminary ceasefire was signed in 2012. Critically, open fighting between the Myanmar army and the main Karen opposition groups had declined over the year leading up to the ceasefire. Data on health and human rights are crucial to inform reconciliation and transitional justice efforts that can ensure that Karen people also benefit from the democratic liberalization and opening economy in Myanmar. This paper reports on results of a cross-sectional survey of human rights violations and health indicators in eastern Myanmar; the data were collected in January 2012 with a recall period coinciding with a period of substantially reduced active conflict in the region.

## Methods

The project was a collaboration between Physicians for Human Rights, The Center for Public Health and Human Rights at Johns Hopkins Bloomberg School of Public Health, and five community-based organizations working in Karen state: Backpack Health Worker Team (BPHWT), Karen Department of Health and Welfare (KDHW), Karen Youth Organization (KYO), the Committee for Internally Displaced Karen People (CIDKP) and one group that wishes to remain anonymous.

**Fig 1:**
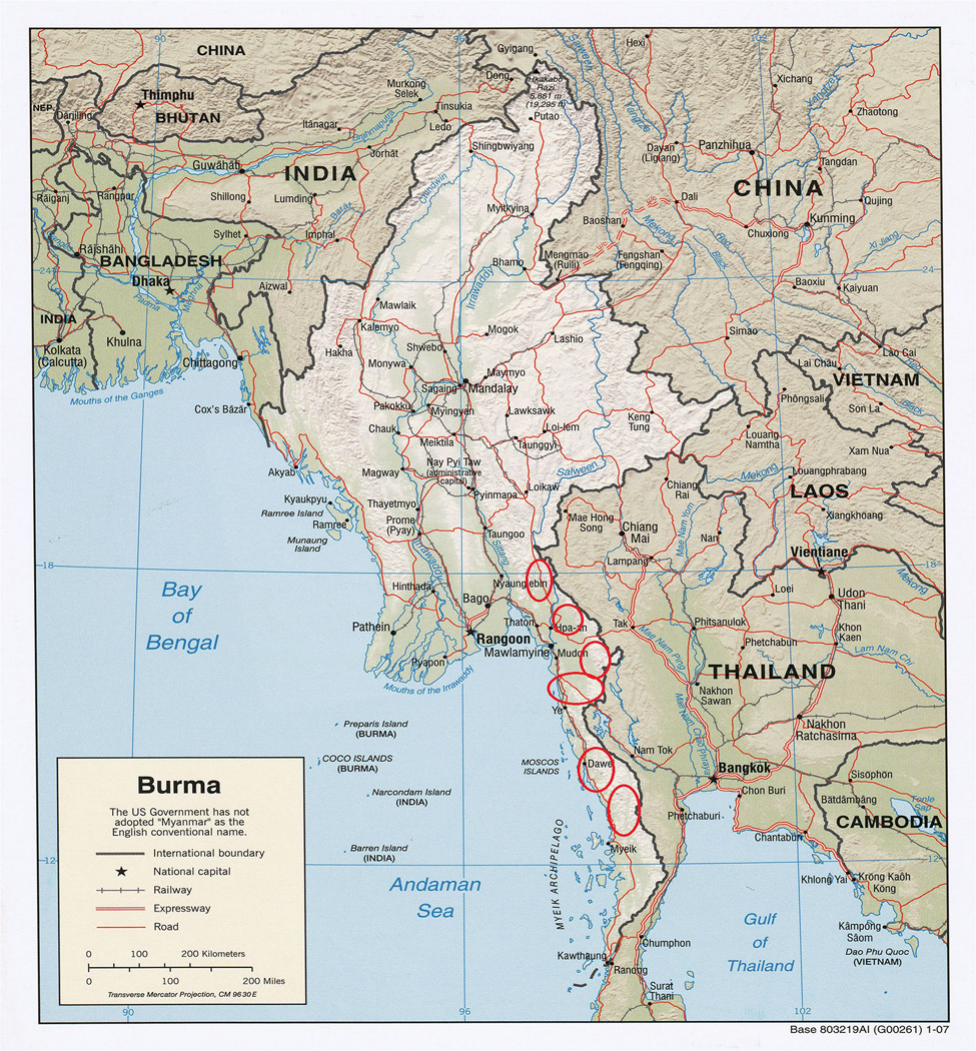
Sampling Frame.

The sampling universe for the cross-sectional survey included adults and children living in clinical catchment areas served by BPHWT and KDHW in Karen state and also adults and children living in three townships around Dawei town in Tanintharyi Region, eastern Myanmar (Fig 1). The sampling frame consisted of approximately 80,000 people living in ~250 villages spread over a region with substantial geographical variation, including jungle-covered mountainous regions and coastal plains with paddy fields.

### Survey Questionnaire

The survey questionnaire was designed to assess human rights violations, access to healthcare, and food security. The survey consisted of four modules: demographic/household listing, access to healthcare, food security and human rights violations each described briefly below.

#### Household Listing and Health Indicators

This module aimed to estimate household size, male to female sex ratio, age distribution, middle-upper arm circumference (MUAC), night blindness and diarrhea of household members. The head of household responded for everyone in the household. If households reported a member who had night blindness or another severe health condition, surveyors referred that person to the nearest clinic. Surveyors listed ethnicity, religion, marital status and occupation of the head of household.

#### Healthcare access

The second module included questions to assess the type, availability and accessibility of health care services in the region.

#### Food Security

The questionnaire incorporated the six-question USAID Food and Nutrition Technical Assistance Project (FANTA) household hunger (HHH) survey and the months of adequate household food production (MAHFP) survey. [14,18,19] We included MAHFP because it has been validated to cover a one-year time frame, which, unlike the HHH, would extend to before the most recent harvest in Karen state. [20] MAHFP is measured by asking the head of household to consider each month in the preceding year and to recall if there was any time during that month in which the household did not have enough food to meet its needs. The number of months in which any single household was able to meet it food requirements is MAHFP.

#### Human Rights Violations

The module covered exposure to armed groups, forced labor, theft or destruction of food, restrictions on movement, displacement and assault. If a respondent said that they had experienced a human rights violation they were asked several follow-up questions to confirm when the event happened, to identify the perpetrator, to identify the family member who experienced the violation and to affirm whether the respondent was an eyewitness or not. Only human rights violations for which a respondent could answer these questions were included in the analysis. In some cases respondents said individual civilians were responsible for the human rights violation; these violations were recoded as “no violation” for the analysis because international law indicates that, typically, only government actors and in some cases non-state armed groups (NSAGs) are considered perpetrators of human rights violations. For the analysis, NSAGs were separated into two groups: those that has signed a ceasefire with the Myanmar government (Karen National Union-Peace Council) and those that had not (Karen National Liberation Army and some breakaway factions of DKBA). Border Guard Force (BGF) includes ethnic armies that signed allegiance to the Myanmar army and operated to some extent under their command, at the time BGF included most of DKBA and other Karen units designated BGF.

### Instrument Development and Training

The content, order, and organization of the modules described above were based on an instrument that had been previously used in Chin State, Myanmar [14], with appropriate modifications for use in Karen state. These adjustments were made via consultation with the partnering CBOs about the content and the wording of the questions to ensure that the survey was capturing important data and that the survey participants would understand the meanings of the questions. The instrument was translated into Sgaw Karen and Burmese and back-translated to English to ensure consistency.

Partner CBOs committed 22 surveyors (16 male, 6 female; age range 20–38 years) who were fluent in Burmese or Sgaw Karen and had knowledge of the terrain, political climate and local leaders in the area where they surveyed. Seven of the surveyors worked with youth groups and the remainder were community health workers. At a central location in Mae Sot, Tak Province, Thailand, surveyors were trained in lectures and practical sessions over two weeks. They were required to pass a final check-out test before they went to the field. The field testing was done in Mae La refugee camp, approximately ~65 kilometres from Mae Sot. The process of training and pre-testing resulted in further refinements to the survey instrument.

### Sample Size and Cluster Selection

We calculated the required sample size (n = 720) to be able to estimate with 5% precision the prevalence of reported human rights violations; assumptions included a maximum prevalence of 15%, a survey return rate of 82% (both estimated from previous surveys in Karen state), and a design effect of 3.0. [21–23] To reach this number, we determined that a 90 x 8 design was most appropriate for this survey, in order to account for the uneven distribution of outcomes measured and to minimize the impact of losing an entire cluster of data if a surveyor lost data forms in the event that he or she had to flee suddenly due to insecurity. [23–30] Partner organizations provided population data for their clinical catchment areas; when data were missing we estimated population size based on the number of houses in the village. We randomly selected villages by assigning probabilities of selection proportional to size and surveyors selected houses in each village using the spin-the-pen technique. [14,31,32]

### Survey Implementation

Surveyors conducted the study during January 2012. The time period covered by the questionnaire was one year prior to the interview, with the exception of the household hunger section (prior month) and health status questions (prior two weeks). Before approaching a village, surveyors assessed the security situation and they sampled the next closest village if they determined there was a security risk. Prior to initiating work within any village, the surveyors first obtained informed consent from the village leader and interviewed the leader about exposure to armed groups and access to health care. The surveyor next selected houses to sample identified the head of household or another adult, obtained informed oral consent for participation and began the interview. Neither the village leader nor heads of household received compensation for participating in the survey. Consent to begin the questionnaire (given by head of household or other adult) and also to measure children’s MUAC (given by parent or guardian) was recorded by marking boxes in the questionnaire; in order to maintain anonymity of participants we did not use written consent. All three ethics committees used for this research approved the consent procedure.

Survey participants were 15 years of age or older if married, 18 years or older if unmarried, living in Karen state, spoke Burmese or Sgaw Karen, displayed sound psychological state to answer sensitive questions and provided informed consent to participate. Children 6 to 59 months old who were residents of the enrolled household whose parent had provided informed consent were included for MUAC measurements. Anyone who ate meals at the house for the two months preceding the survey was considered a household member.

### Data Analysis

The goal of the data analysis was to identify associations between human rights violations and health outcomes. All analyses were performed using STATA 13 and svy commands to apply Taylor linearization to the data to adjust for cluster sampling. Data was weighted at either the clinical area level or the village level by population before analyses were performed. Survey coverage, participation rates, and demographic characteristics of the population were estimated, along with prevalence of health outcomes, human rights violations and alleged perpetrators and 95% confidence intervals were calculated.

We used the generalized linear estimation approach to conduct binomial regression with a log link functions in order to estimate risk ratios for human rights violations and health outcomes; in the case of non-convergence we used Poisson models. This approach yields a prevalence rate ratio (PRR), a measure of risk. Household hunger was coded into moderate/severe and none/mild categories, MUAC scores for children under 5 were separated into moderate/severe (<12.5mm) and none/mild (>12.4mm), (based on WHO criteria), and diarrhea and night blindness were recoded as binary variables (present/ not present). We coded MAHFP into a binary variable using 9 months as a cutoff. The distribution of MAHFP peaked at 8 months in households that had experienced human rights violations (HRVs) and at 10 months for households that had not, and this cutoff best captured the difference between the two populations.

About 7% of data for diarrhea and night blindness were missing. We assumed that data were “missing at random” and used multiple imputation with chained equations to estimate values for missing variables (diarrhea, night blindness, human rights violations). [33] Results from the complete case analysis and analysis with imputed data were similar, and only results from the complete-case data set are reported here.

### Ethics Statement

The Physicians for Human Rights (PHR) Ethical Review Board, the Institutional Review Board at the Johns Hopkins Bloomberg School of Public Health and a Karen community advisory team reviewed and approved the research plan.

## Results

Surveyors approached 90 villages throughout the target area. They encountered security risks in 10 villages and substituted 8 for a total of 88 village leaders approached. One village leader refused consent, so 87 total villages were sampled. Within these 87 villages, 696 households were approached by the surveyors, with 686 (98.6%) consenting to participate.

### Demographics

The 686 households sampled represented a total of 3657 people (mean household size 5.3, range 1 to 16). Only a small number (42, 6.2%) of households were female-headed. The male-female ratio for 15–25 year olds was 0.88 and that for 15–45 year olds was 0.93. The population was 13.4% Christian and 59.5% Buddhist, with atheist, animist, “other” and “no response” making up the remaining 7.1%. Seventy-five percent of respondents said they were from the Sgaw Karen ethnic group and 4.4% reported they were Po Karen. Over two thirds of people interviewed were married and most said they were farmers.

### Health

Diarrhea prevalence in children was 14.2% and in everyone it was 5.8% (Table 1). Night blindness overall was 4.4%, in women of child-bearing age it was 5.6% (Table C in S1 File.). Of 423 children aged 6 to 59 months, three (1.0%) had MUAC less than 11.5mm, 10 (3.2%) had MUAC between 11.4 and 12.5, and 24 (8.6%) had MUAC between 12.4 and 13.5 (Table B in S1 File). Surveyors reported difficulty in locating and measuring children, and 108 (24.8%) of eligible children were not measured for MUAC.

**Table 1:** Diarrhea.

	Everyone		0–59 months	
	yes	%	yes	%
None	3164	86.5	345	81.6
Yes	212	5.8	60	14.2
Missing	281	7.7	18	4.3
Total	3657		423	

**Fig 2:**
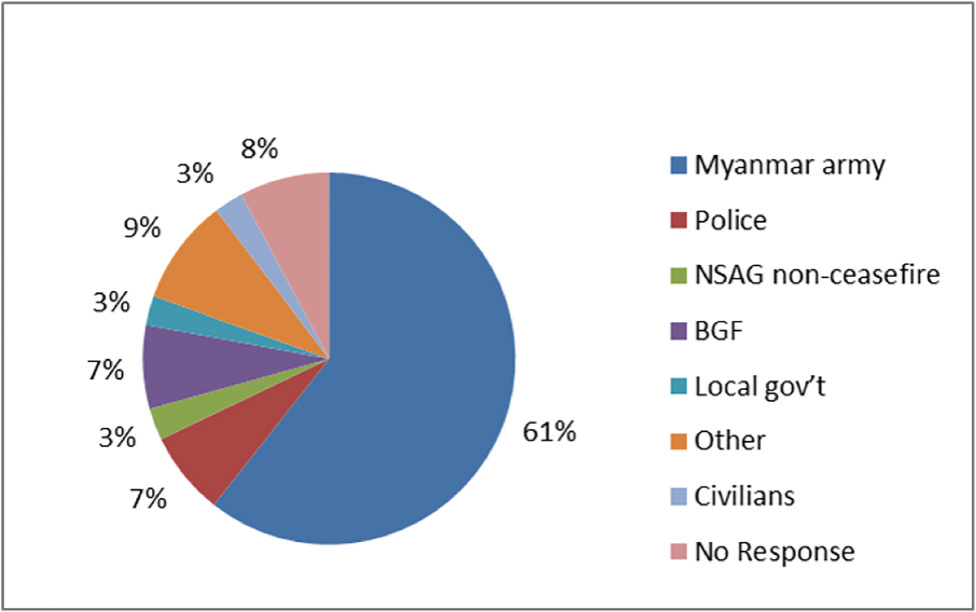
Percent of Households Reporting Sufficient Food in Each Month of 2011.

Analysis of household hunger questions indicated that 581 (84.7%) of households had low household hunger, the lowest possible rank on this scale, 85 (12.3%) had moderate hunger and six households (0.9%) had severe hunger (total moderate/severe: 13.2%, Table 2). Female-headed households experienced household hunger in similar proportions: 36 (87.8%) had low hunger, while 5 (12.2%) had moderate/severe hunger. In 2011 the proportion of households reporting not having enough food to meet their needs ranged from 19.9% in December to 47.0% in September, which is just before the harvest (Fig 2). Two hundred eighty eight (43.3%) households reported having adequate food for every month in 2011. The remainder reported at least one month of inadequate food production, with 99 (14.9%) households reporting that they were not able to meet their food needs for any month in 2011 (Table A in S1 File).

**Table 2:** Household Hunger.

	All Households		Female-Headed Households	
	n	%	n	%
**Severe HHH**	6	0.9	1	2.4
**Moderate HHH**	85	12.3	4	9.8
**Low HHH**	581	84.7	36	87.8
**Moderate or severe HHH**	91	15.8	5	14.3

### Human Rights Violations

**Fig 3:**
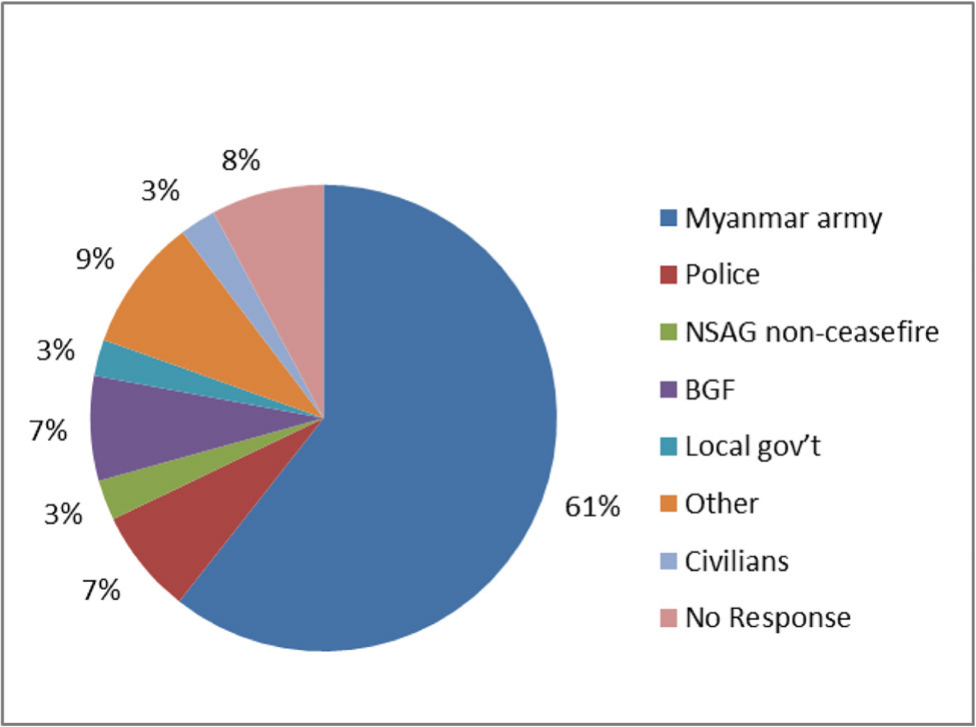
Perpetrators of Human Rights Violations.

In 2011, 210 households (30.6%) surveyed reported experiencing at least one human rights violation. Forced labor was the most common violation (185, 24.9%) and included being forced to carry supplies for an armed group, to sweep for mines, to grow crops, to work for the military or other forced labor. Eleven households (1.4%) reported any kind of assault, including kidnapping, rape, torture and beating. Eighty six households (14.9%) experienced at least two human rights violations, and one household reported six violations (Table 3). In most cases, the Myanmar army or other government officials were responsible for the violation (Fig 3).

**Table 3:** Human Rights Violations.

Type of Violation	Households responding	Cases in 2011	%^a^	95% CI lower	95% CI upper	% missing data^b^
Forced to be porters	672	90	14.4	9.9	20.5	2
Forced to sweep for mines	672	5	0.5	0	1.5	2.8
Forced to grow crops	670	25	2.8	1.4	5.6	3.1
Forced to work for military	556	50	9.5	6.1	14.5	31
Other forced labor^c^	671	91	14.1	9.7	20.1	3.1
Blocked from accessing land	663	20	4.3	2.2	8.1	3.9
Food stolen or destroyed	681	24	4.1	3.4	9.8	0.8
Restricted movements	670	28	6	3	11.3	0.7
Religious discrimination	662	6	0.9	0.4	2.3	2.8
Kidnapped	685	1	0.2	0	1.1	0.2
Wounded	675	1	0.2	0	1.7	2.6
Tortured	673	9	1.3	0.1	2.8	2.5
Sexually Assaulted	671	5	0.1	0	2.8	3.3
Any forced labor	684	185	24.9	19.1	31.6	0.3
Any assault	685	11	1.4	0.6	3.1	1
Any HRV	686	210	30.6	22.7	35.6	0
No HRVs	686	476	71.3	64.4	77.3	0
Only one HRV	686	127	14.4	11.5	17.9	0
Two HRVs	686	42	6.9	4.3	10.9	0
Three HRVs	686	29	5.2	3.1	8.6	0
Four HRVs	686	6	1.1	0.4	2.8	0
Five HRVs	686	3	0.6	0.2	2	0
Six HRVs	686	3	0.5	0.1	2.1	0

^a^ calculated using Taylor linearization, so percents may not match a direct calculation

^b^ includes refused to answer, not recorded

^c^ includes cutting wood or bamboo, cleaning compounds, roads, building bridges or buildings

Table 4 shows substantially higher risk for a household member to have diarrhea in household that had experienced any forced labor (PRR 2.63, 95% CI 1.94 to 3.55), any human rights violation (PRR 2.73, 95% CI 1.96 to 3.80), forced portering (PRR 2.32, 95% CI 1.50 to 3.58), theft or destruction of food (PRR 2.10, 95% CI 1.11 to 3.98), restricted movement (PRR 2.61, 95% CI 1.19 to 5.71), or multiple human rights violations (PRR 2.67, 95% CI 1.67 to 4.28). Similarly, children under 5 were more likely to have diarrhea if their household experienced theft or destruction of food (PRR 2.91, 95%CI 1.1–3.09), restrictions on movement (PRR 2.61, 95%CI 1.19–5.71) or multiple human rights violations (PRR 2.67. 95% CI 1.67–4.28). Household hunger was associated with theft or destruction of food (PRR 2.59, 95% CI 1.38 to 4.88) and being blocked from accessing land (PRR 2.18, 95% CI 1.03–4.61), while inadequate food production was associated with forced labor (PRR 1.81, 95% CI 1.43 to 2.31), forced portering (PRR 2.03, 95% CI 1.58 to 2.62), restricted movement (PRR 1.72, 95%CI 1.14–2.59), any human rights violation (PRR 1.93, 95% CI 1.50 to 2.46), and multiple human rights violations (PRR 1.96, 95% CI 1.50 to 2.56).

**Table 4:** Associations Between Human Rights Violations and Health Outcomes.

Human Rights Violation	Health Outcomes											
	Diarrhea			Diarrhea in children under 5			Household hunger			MAHFP		
	PRR	95 low	95 high	PRR	95 low	95 high	PRR	95 low	95 high	PRR	95 low	95 high
**Any forced labor**	2.63	1.94	3.55	1.44	0.9	2.31	1.25	0.76	2.08	1.81	1.43	2.31
**Any HRV**	2.73	1.96	3.8	1.51	0.93	2.47	1.39	0.85	2.29	1.93	1.5	2.46
**Forced to porter**	2.32	1.5	3.58	0.9	0.42	1.92	1.24	0.65	2.36	2.03	1.58	2.61
**Forced to grow crops**	0.52	0.21	1.33	0.42	0.04	4.19	-	-	-	1.02	0.56	1.84
**Other forms of forced labor**	1.04	0.59	1.84	0.56	0.16	1.93	0.72	0.36	1.46	2.25	1.77	2.86
**Blocked from accessing land**	1.36	0.61	3	1.24	0.24	6.38	2.18	1.03	4.61	1.47	0.86	2.5
**Food stolen or destroyed**	2.1	1.11	3.98	2.91	1.58	5.34	2.59	1.38	4.88	0.71	0.4	1.28
**Restricted movement**	2.61	1.19	5.71	3.2	1.74	5.88	1.98	0.73	5.35	1.72	1.14	2.59
**Two or more HRVs**	2.67	1.67	4.28	1.53	0.64	3.63	1.69	0.93	3.1	1.96	1.5	2.56

Adjusted for household size, type of water supply, clinical catchment area, religion, topography, female-headed households and exposure to any other human rights violations.

## Discussion

Almost one third of households in Karen State reported experiencing one or more human rights violations in 2011, and these violations were statistically associated with higher risk of diarrhea, household hunger and household food production. These results provide further evidence decrease in conflict in recent years in Myanmar has not led to concomitant reductions in human rights violations at the population level.

The prevalence of human rights violations in this survey is less than what has been reported in Karen state in 2004 but greater than what was reported in 2007. The 2004 survey found that 25.2% of respondents reported that the military stole or destroyed their food, 8.9% reported forced displacement, 2.1% were physically attacked and 32.6% of respondents reported forced labor; in total 52.1% of respondents reported having experienced at least one human rights violation. [12,34] A 2007 survey conducted in more stable areas suitable for a maternal and child health research project found that 1.2% of respondents reported that their fields were attacked, 3.1% reported having livestock stolen, 1.9% reported that their food was taken by the army, 1.5% reported forced labor, and 10.5% reported forced displacement. [5]

The results of our survey indicate that human rights abuses continued to be systematic and widespread in Karen state in 2011. We measured a lower prevalence of assaults compared with previous years, but a similar prevalence of forced labor. These findings might be explained because there was less conflict in Karen state in 2011 than in 2004 or 2007. Although there was limited fighting in 2011, the Myanmar army maintained over 250 outposts or bases in Karen state. [35] Army policies dictate that battalions supply themselves from the area they are assigned to patrol, and this often results in forced labor and theft of food from civilians. Our data indicate that militarization in Karen state, even in the near absence of fighting, can result in human rights violations and can adversely impact the health and food security of civilian populations.

Household food insecurity was common. Data for HHH and MAHFP have not previously been collected in Karen state, but a June 2010 survey found that only half of households surveyed had enough food stored to last until the harvest in November. [35] This is consistent with our finding that in September 2011, 47.0% of households had insufficient food. The cross-sectional prevalence of global acute malnutrition among children (GAM, as measured by MUAC) was lower in this survey (4.2%) than what has been reported previously in eastern Myanmar. Previous nutrition surveys conducted in Eastern Myanmar in have estimated the prevalence of GAM was 12.6% in 2004, 14.8% in 2009 and 11.3% in 2013. [36] Several factors may have contributed to a lower prevalence of acute child malnutrition in our survey, including information bias (one-quarter of children lacked MUAC measurements) and the timing of the survey immediately after the harvest when most households had sufficient food supplies. We are not certain why so many children lacked MUAC measurements; it is possible that they were not at home during the time of the survey, as it is common for people to visit relatives in different villages. Logistical limitations precluded an assessment of chronic malnutrition (stunting), which may have been able to capture persistent effects of malnourishment in early childhood.

Prevalence of child diarrhea and night blindness reported by our survey were higher than the average for southeast Asia. [36] Although Karen state has a history of food insecurity that could account for high prevalence of night blindness, it is also possible that heads of household misdiagnosed night blindness among other household members.

Food insecurity can be related to human rights violation through several mechanisms. Restrictions on movement may prevent farmers from working on their land or trading their crops, and forced labor consumes time that otherwise might have been spent working in fields or to produce money to buy food. This survey identified statistically significant associations between human rights violations and household hunger, months of adequate household food production, diarrhea and night blindness. Surveys in Karen state in 2004 and 2006 also identified associations between food destruction and mortality, food destruction and child malnutrition, forced labor and mortality, and anemia and food security violations. [5] A 2011 report from Chin state, western Myanmar identified associations between household hunger and forced labor, assault, and human rights violations related to food security. [14] These findings are consistent with our findings that human rights violations are associated with food insecurity.

## Limitations

Limitations inherent to cluster sampling all apply to this survey and have been discussed in detail elsewhere. [22,23,27,28] This survey was done in areas of Karen state where community-based organizations are operating and as it did not cover the entire state, the results are not necessarily generalizable to other parts of the state. In the past these organizations tended to focus on areas of conflict, and during the time of the survey most the sampled population was under mixed administration. The age structure of the population was indicative of a population living in an area of conflict, and age structure and proportions of religious and ethnic groups were similar to those reported elsewhere in Karen state. [12,37]. Because of the history of conflict and displacement of the sampled population, it might be more vulnerable in terms of food security and access to healthcare than people living elsewhere in the state.

The surveyors had extensive knowledge and work experience in the area, and per security protocols, they could decide to skip a cluster if they felt it was not safe to work there. Surveyors skipped ten such clusters, reporting that they did so in all cases because Myanmar army or Border Guard Force soldiers were present in these villages, and the surveyors were concerned about their own physical safety. Surveyors reported two cases of having to wait several days until the Myanmar army vacated villages before they could perform the survey. They also reported extensive Myanmar army troop movements and occasional mortaring during the movements that created difficulties for surveyors to travel, especially on roads. We cannot determine if there were systematic differences in human rights violations or health outcomes between the villages that were skipped and those that were sampled. Such differences, if present, would likely result in in an underestimation of the associations between HRVs and health outcomes.

Although surveyors lived and worked in the areas they were assigned to cover, it is possible that interviewees were not comfortable discussing sensitive issues such as health or human rights violations. Due to logistical constraints, we did not match surveyors and respondents by sex. During the informed consent process, surveyors assured respondents of anonymity and confidentiality and it is possible that sensitive information was underreported.

MUAC and household hunger results represent a snapshot of the yearly cycle of malnutrition in Karen state. Rice is harvested from September to November in this area and at the time of the survey (January) it is likely that families were at one of their most food-secure times during the year. A month-by-month analysis of MAHFP confirms this. The nutrition data likely represent a best-case scenario for families over the course of the year.

## Conclusion

Decades of war and human rights violations have taken a toll on health of civilians in Karen state, and political changes that began in the central part of Myanmar in 2010–2011 have been slow to reach this area. Although a conflict had decreased in Karen state at the time of this study, this has not meant an end to human rights abuses or to improved health in the area.

## Supporting Information

S1 File Table A, Months of Adequate Household Food Production. Table B, Malnutrition in Children 6–59 Months. Table C, Prevalence of Night Blindness.
(DOCX)
