# Supplementary material for: Health and Human Rights in Karen State, Eastern Myanmar
Source: PLoS One. 2015 Aug 26;10(8):e0133822. doi: 10.1371/journal.pone.0133822 (PMC4550474; doi:10.1371/journal.pone.0133822)
Supplement: S1 File — Table B, Malnutrition in Children 6–59 Months. Table C, Prevalence of Night Blindness. (DOCX) [file pone.0133822.s001.docx]

**S1 File.**

**Table A. Months of Adequate Household Food Production**

| Month in 2011 | HH had enough food | HH had not enough food | Total responding | % reporting not enough food |
| --- | --- | --- | --- | --- |
| January | 531 | 138 | 669 | 20.6 |
| February | 531 | 138 | 669 | 20.6 |
| March | 525 | 144 | 669 | 21.5 |
| April | 522 | 147 | 669 | 22 |
| May | 517 | 153 | 670 | 22.8 |
| June | 499 | 168 | 667 | 25.2 |
| July | 459 | 210 | 669 | 31.4 |
| August | 415 | 254 | 669 | 38 |
| September | 355 | 315 | 670 | 47 |
| October | 366 | 302 | 668 | 45.2 |
| November | 477 | 191 | 668 | 28.6 |
| December | 534 | 133 | 667 | 19.9 |
| >9 months | 378 | 287 | 665 | 43.2 |

**Table B. Malnutrition in Children 6-59 Months**

| WHO classification | n | % |
| --- | --- | --- |
| Severe | 3 | 1 |
| Moderate | 10 | 3.2 |
| At-risk | 24 | 8.6 |
| No risk | 278 | 91.4 |

**Table C. Prevalence of Night Blindness**

|  | Everyone | | 0-59 months | | Women 15-45 years | |
| --- | --- | --- | --- | --- | --- | --- |
|  | yes | % | yes | % | yes | % |
| None | 3238 | 88.5 | 402 | 95 | 764 | 85.4 |
| Yes | 155 | 4.2 | 3 | 0.7 | 50 | 5.6 |
| Missing | 264 | 7.2 | 18 | 4.3 | 81 | 9.1 |
| Total | 3657 |  | 423 |  | 895 |  |
